# Chromosome-level genome assembly of grass carp (*Ctenopharyngodon idella*) provides insights into its genome evolution

**DOI:** 10.1186/s12864-022-08503-x

**Published:** 2022-04-07

**Authors:** Chang-Song Wu, Zi-You Ma, Guo-Dong Zheng, Shu-Ming Zou, Xu-Jie Zhang, Yong-An Zhang

**Affiliations:** 1 State Key Laboratory of Agricultural Microbiology, College of Fisheries, Huazhong Agricultural University, Wuhan, China; 2 Genetics and Breeding Center for Blunt Snout Bream, Key Laboratory of Freshwater Aquatic Genetic Resources, Ministry of Agriculture, National Demonstration Center for Experimental Fisheries Science Education, Shanghai Ocean University, Shanghai, China; 3 Engineering Research Center of Green Development for Conventional Aquatic Biological Industry in the Yangtze River Economic Belt, Ministry of Education, Wuhan, China; 4 Hubei Hongshan Laboratory, Wuhan, China; 5 Laboratory for Marine Biology and Biotechnology, Qingdao National Laboratory for Marine Science and Technology, Qingdao, China

**Keywords:** Grass carp, Cyprinid fish, Chromosome-level genome, Comparative genomics, Adaptive evolution

## Abstract

**Background:**

The grass carp has great economic value and occupies an important evolutionary position. Genomic information regarding this species could help better understand its rapid growth rate as well as its unique body plan and environmental adaptation.

**Results:**

We assembled the chromosome-level grass carp genome using the PacBio sequencing and chromosome structure capture technique. The final genome assembly has a total length of 893.2 Mb with a contig N50 of 19.3 Mb and a scaffold N50 of 35.7 Mb. About 99.85% of the assembled contigs were anchored into 24 chromosomes. Based on the prediction, this genome contained 30,342 protein-coding genes and 43.26% repetitive sequences. Furthermore, we determined that the large genome size can be attributed to the DNA-mediated transposable elements which accounted for 58.9% of the repetitive sequences in grass carp. We identified that the grass carp has only 24 pairs of chromosomes due to the fusion of two ancestral chromosomes. Enrichment analyses of significantly expanded and positively selected genes reflected evolutionary adaptation of grass carp to the feeding habits. We also detected the loss of conserved non-coding regulatory elements associated with the development of the immune system, nervous system, and digestive system, which may be critical for grass carp herbivorous traits.

**Conclusions:**

The high-quality reference genome reported here provides a valuable resource for the genetic improvement and molecular-guided breeding of the grass carp.

**Supplementary Information:**

The online version contains supplementary material available at 10.1186/s12864-022-08503-x.

## Background

Grass carp has a breeding history of more than 1700 years in China. Since the 1980s, grass carp has been introduced directly or indirectly to various countries of the world, such as the United States, Mexico, India and Hungary [1]. Its artificial breeding began in 1958 and it is the most productive species in freshwater fish farming in the world, with great economic effects, providing a large amount of high-quality protein and trace elements for all mankind. In 2020, the total production of freshwater fish farming in China was 30.89 million tons, of which grass carp had the highest production (5.57 million tons), accounting for about 18% of the total production [2]. Due to strong adaptability, rapid growth, and large size of grass carp, it is known as one of the “Four Domesticated Fish” in freshwater culture in China, together with the bighead carp (*Hypophthalmichthys nobilis*), silver carp (*Hypophthalmichthys molitrix*), black carp (*Mylopharyngodon piceus*). It should be noted that the “Four Domesticated Fish” belong to the family Xenocyprididae in the latest classification system (Eschmeyer’s Catalog of Fishes), which previously classified them as Cyprinidae. Grass carp is a typical herbivorous fish and mainly distributed in the Yangtze, Pearl and Heilongjiang rivers in China. Food habit transition during the development of grass carp facilitates the rapid growth and development. Previous research has shown that the body weight, body length and intestine length of transitioned grass carp are significantly higher than that of the untransitioned grass carp. The genes involving circadian rhythm, lipid synthesis and metabolic pathways have undergone adaptive changes after transition of food habits, making it more effective to use the nutrients in plants [3].

Via high-throughput whole-genome sequencing technology we can accurately obtain the base sequences of a species to decipher its genetic information. It can reveal the complexity and genetic diversity of the species genome, which brings new research methods and solutions to explore the mechanism of species development and environmental adaptability, thereby speeding up the breeding process of new varieties [4, 5]. In recent years, the genome sequences of major freshwater economic fishes in China have constantly published. Such as blunt snout bream (*Megalobrama amblycephala*) [6–8], goldfish (*Carassius auratus*) [9], common carp (*Cyprinus carpio*) [10], silver carp and bighead carp [11]. These genomic data not only provide convenience for studying the biological problems of this species, but also lay the foundation for the mining of functional genes, the selection of excellent traits and the breeding from an evolutionary perspective for other species. The genome sequence of grass carp has been published in 2015 [3]. However, the continuity level of the sequence based on second-generation sequencing is low, and it is not assembled to the chromosome level. Here, we report a high-quality chromosome-level genome sequence of grass carp, assembled using Hi-C chromatin interaction maps and PacBio long reads. The majority of the assembled sequences were anchored into 24 scaffolds, consistent with the 2n = 48 karyotypes of grass carp. Large-scale RNA sequencing data was generated from twelve tissues of grass carp to assist the prediction of protein-coding genes. In addition to perform the comparative and evolutionary studies of grass carp and other 9 cyprinid fishes using the genome sequences, we also identified genes and regulatory elements that may be related to grass carp herbivorous traits and unique body plan. The high-quality chromosome-scale genome sequence lays a solid foundation for the study of the genetic characteristics of grass carp, and for the understanding of genome evolution in teleosts more generally.

## Results

### Genome sequencing and assembly

**Fig. 1:**
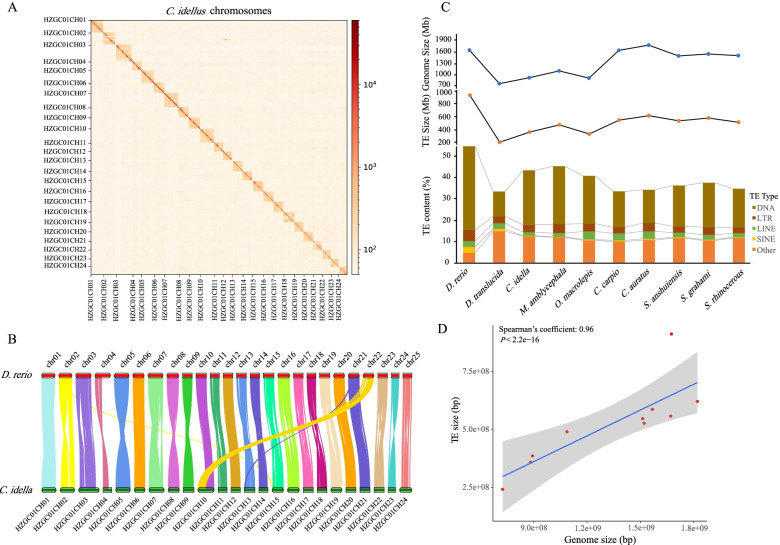
Structural characteristics and evolution of cyprinid genomes. **A** Chromosomal contact maps of grass carp using Hi-C data. The blocks refer to the contacts between one location and another. The deeper colors represent the higher intensity of contact. **B** Collinear blocks of genes shared by the zebrafish and grass carp genomes. Each colored line represents a best match between the two species. The number of genes in blocks is greater than 30. **C** The average genome sizes, TE sizes, and contents of different TE types of cyprinids. **D** The relationship between genome size and TE size in cyprinids using the spearman method

The grass carp genome consists of 24 pairs of chromosomes (2n = 48) [3]. To acquire high-quality genome assembly, the single-molecule real-time PacBio long reads (156.3 Gb, 175×, N50 19.8 Kb) were used. We first generated a contig-level assembly, and then organized them into scaffold-level genome assembly using Hi-C reads (~ 139.6 Gb). This was followed by extensive manual management using LACHESIS and the Hi-C maps to remove mis-joins and redundant contigs to generate a high-quality genome assembly. De novo assembly of PacBio long reads combined with error correction using BGI platform short reads (117.8 Gb) as well as contaminant and redundancy removal resulted a final genome sequence of grass carp that had a total length of 893.2 Mb, and contained 180 contigs and 30 scaffolds with N50 lengths of 19.3 Mb and 35.7 Mb, respectively. A total of 891.9 Mb (99.85%) of the assembly sequences were organized into 24 pseudo-chromosome groups (each > 25 Mb) (Fig. 1A), consistent with the commonly observed *n* = 24 karyotypes of grass carp. Compared with the previously released grass carp genome assembly [3], the N50 length of the current genome scaffolds and contigs were much longer (Table S2). These scaffolds are hereafter referred to as chromosomes. Based on assembled high-quality genome sequence, we performed the heterozygosity analysis. The heterozygosity ratio was approximately 0.1% (926,251 SNPs), thus suggesting a simple genome of gynogenetic grass carp. Then, based on de novo, homolog-based, and RNA-seq approaches, 30,342 protein coding genes were identified in grass carp, among which 28,963 genes were detected to be expressed in twelve tissues. We also used the NR database for functional annotation of the 30,342 genes, and 29,509 genes were aligned. The distribution density of genes in chromosomes was calculated using RIdeogram [12]. The results showed that the third chromosome (HZGC01CH03) is the longest (~ 58 Mb) and contains the most genes (2243 genes) (Fig. S1). Searches for BUSCO using the actinopterygii_odb9 core gene sets showed that the assembly genome contained 95.7% of the complete sequences and 2.5% of the partial sequences of genes, with 1.8% of the genes missing. Moreover, 7.3% of the genes were identified as duplicates (Table S3). Alignment of genes showed highly synlinear between grass carp and zebrafish, and up to 25,790 grass carp genes (85% of the 30,342 total genes) were located on syntenic blocks (Fig. 1B, Fig. S2). Same as previous research, the zebrafish chromosomes 10 and 22 recombine and integrate into grass carp chromosome 10 [3], which explains that zebrafish has 25 pairs of chromosomes and grass carp has only 24 pairs.

The grass carp genome showed an overall repeat content of 43.26%, which was similar to the 45.68% for blunt snout bream [6, 7], higher than that for many of the sequenced teleost genomes [32.65% in *O. latipes* [13] and 34–38% in cave fish [14]], but much lower than that of the zebrafish (54.47%) [15] (Table S1). Among the published genomes of cyprinid fish, the size of the genome is quite different. The smallest genome (*D. translucida*) is ~ 0.73 Gb [16], and the largest genome (*C. auratus*) is ~ 1.8 Gb [9] (Table S1). Repeat sequence analysis confirmed that transposable element (TE) content is the major cause of genome size variation (Spearman’s coefficient: 0.96, *p* value < 2.2e− 16), the size of TEs ranges from 241.9 Mb (*D. translucida*) to 912.2 Mb (zebrafish) (Fig. 1C, D). These further results suggested that the explosion of DNA, but not LTR, LINE and SINE elements, greatly contributed to the evolution of the large genome of cyprinid fish.

### Phylogenetic tree analysis

**Fig. 2:**
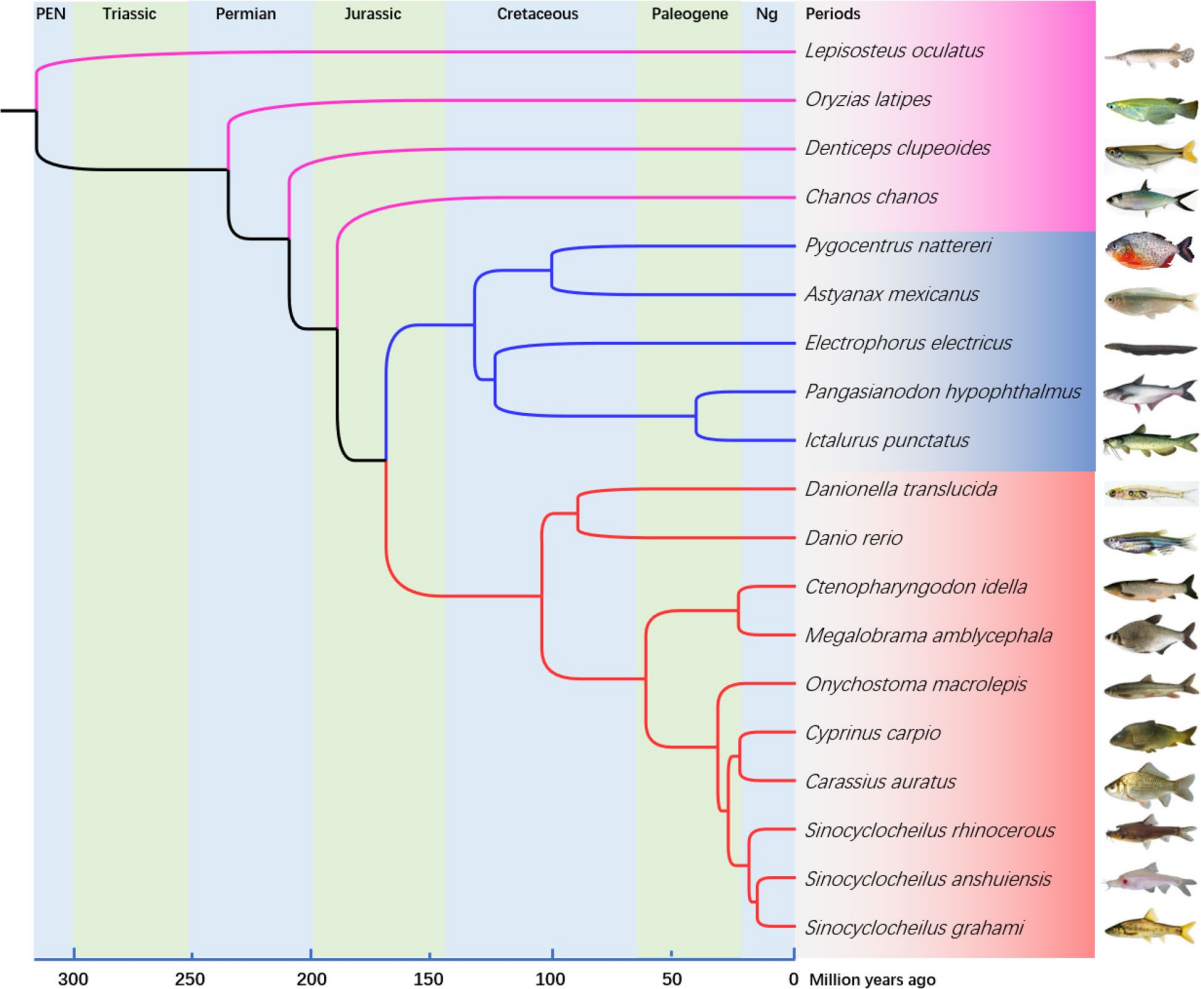
The maximum likelihood phylogenetic tree from single-copy gene protein sequences of 19 teleosts. To compute the node supports, 1000 bootstraps were used, and all nodes have 100% support. The red marker represents cyprinids, blue marker represents the sister group of cyprinids, purple marker represents outgroup. (PEN: Permian, Ng: Neogene)

A phylogenetic tree was constructed using 5067 single-copy orthologous genes from 19 teleost fishes (Fig. 2). To ensure that the reconstructed phylogeny is robust in the influence of incomplete lineage sorting (ILS), we performed several analyses with the coalescent-based phylogenetic methods ASTRAL [17] and MP-EST [18] using the single-copy orthologous genes. The topology of the new obtained ASTRAL (Fig. S3A) and MP-EST (Fig. S3B) coalescent trees are identical to the phylogenetic tree (Fig. 2). Using fossil calibrations [19], we estimated the emergence of cyprinid fish at ~ 100 million years ago (Mya) (mid Cretaceous), and the emergence of grass carp at ~ 23 Mya (late Paleogene).

### Gene family analysis

**Fig. 3:**
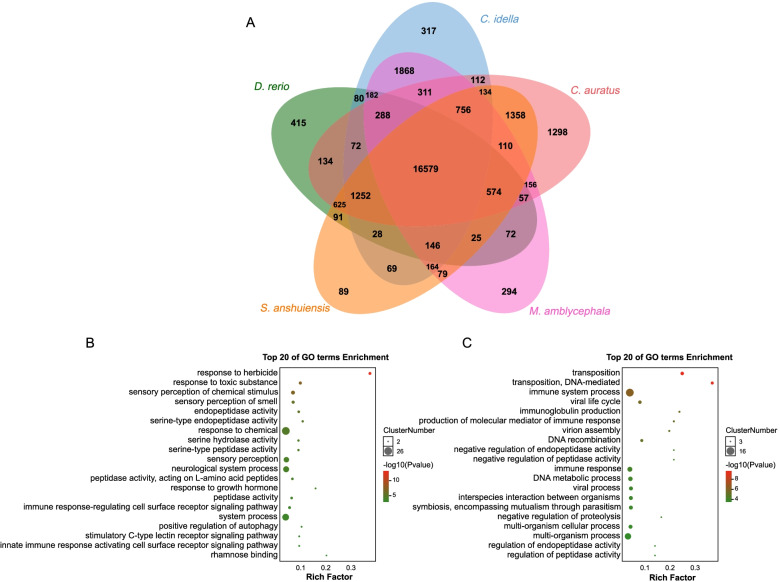
Venn diagram of gene families among five cyprinids and functional enrichment analysis of gene families specific to zebrafish and grass carp. **A** Common and unique gene families among five cyprinids shown with a Venn diagram. **B** GO enrichment analysis of gene families specific to zebrafish. **C** GO enrichment analysis of gene families specific to grass carp

Gene families were defined among 5 selected cyprinid fishes (*D. rerio*, *C. idella*, *M. amblycephala*, *C. auratus* and *S. anshuiensis*) in the present study. In total, 27,735 gene families and 23,518 orthologous families (at least existing in two species) were identified in the 5 selected species. The results showed 16,579 gene families that commonly exists in all 5 species (Fig. 3A). One thousand eight hundred sixty-eight gene families were identified as specific ones in grass carp and blunt snout bream which are considered to be herbivorous cyprinid fish [3, 7]. GO terms were enriched within the 1868 gene families, such as DNA recombination, DNA integration and DNA-mediated transposition (Table S4). In addition, zebrafish and grass carp have 415 and 317 unique gene families, respectively. GO enrichment results showed that zebrafish unique gene families are mainly related to nervous system (sensory perception of chemical stimulus, sensory perception and neurological system process), immune system (immune response-regulating cell surface receptor signaling pathway, immune response-regulating signaling pathway and activation of immune response) (Fig. 3B). Interestingly, GO enrichment analysis of grass carp unique gene families has also enriched GO terms related to DNA recombination, such as DNA-mediated transposition, DNA recombination and transposition (Fig. 3C). Notably, two gene families are related to pheromone (*p* value < 0.01), including *vmn2r26* (5 genes) and *vmn2r1* (2 genes) for grass carp unique gene families. Besides, we also predicted 974 newly evolved genes of grass carp [3768 unique genes were detected in grass carp, combined with the transcriptome data of 12 tissues, if a gene was expressed in 12 tissues and the expression level TPM (Transcripts Per Million) ≥ 20, we consider this gene to be a newly evolved gene in grass carp] [20], including immune system (25 genes), nervous system (8 genes), digestive system (4 genes), etc. (Fig. S4).

### Expansion and contraction of gene families

**Fig. 4:**
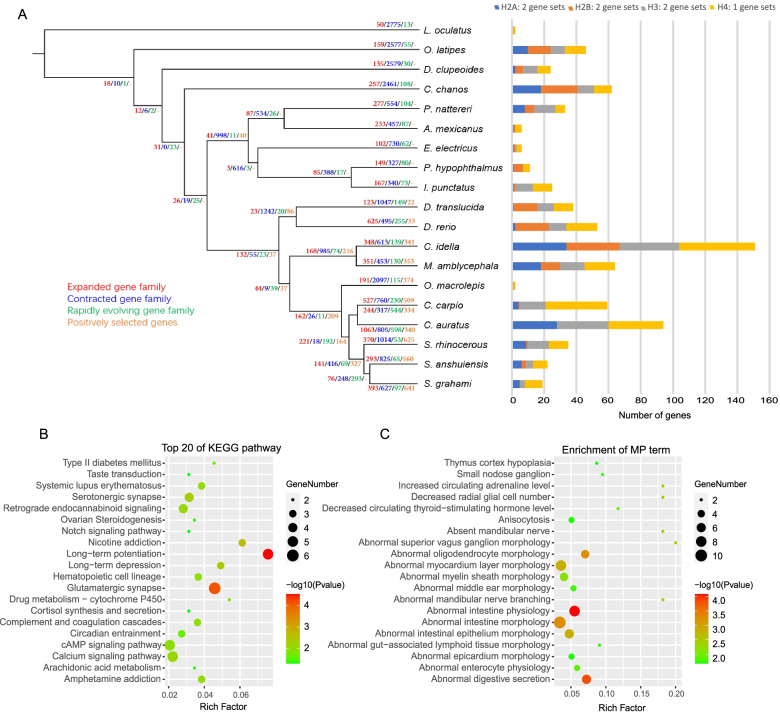
Expanded gene families, contracted gene families, rapidly evolving gene families and positively selected genes (PSGs) among teleosts and functional enrichment analysis. **A** PSGs, rapidly evolving gene families, expanded and contracted gene families are shown along the phylogenetic tree (left). The number of four rapidly evolving histone genes in 19 teleosts (right). **B** KEGG enrichment analysis of expanded gene families in 10 cyprinids. **C** MP enrichment analysis of PSGs in grass carp

To study the expanded gene families in cyprinid fish, we performed expansion and contraction analysis of gene families, and identified 132 gene families expanded in the cyprinid fish (Fig. 4A). One hundred sixty-eight gene families expanded in grass carp and blunt snout bream. The top 20 pathways of KEGG enrichment indicated that expanded gene families mainly related to immune system and diseases, such as hematopoietic cell lineage, antigen processing and presentation, Th1 and Th2 cell differentiation and systemic lupus erythematosus and so on (Table S5). We further studied whether the grass carp showed unique changes in gene families and identified 348 expanded gene families and 613 contracted gene families in this species. The enrichment results indicated that the function of the expanded gene families is mainly related to immune system (Table S6, Table S7), however, DNA integration term (7 gene families, *p* value = 7.54e-05) was enriched again. Interestingly, we identified 139 rapidly evolving gene families in grass carp, of which 7 gene families belong to histones (Fig. 4A right). KEGG enrichment analysis of the expanded gene families in the cyprinid fish indicated that nervous system was mainly enriched, such as long-term potentiation, glutamatergic synapse, long-term depression, serotonergic synapse and retrograde endocannabinoid signaling (Fig. 4B).

### Positive selection analysis

We obtained a high-confidence orthologous gene set for the 19 teleosts using *L. oculatus*, *O. latipes*, *D. clupeoides* and *C. chanos* as outgroups. Using the resolved phylogeny of teleosts, we identified positively selected genes (PSGs). Thirty-seven PSGs in cyprinid fish branch were detected, such as *mag* (myelin associated glycoprotein) and *ptprm* (protein tyrosine phosphatase receptor type M), which have been shown to play an important role in the development of the nervous system [21, 22]. Pancreatic ribonuclease is encoded by the *rnase1* gene and is an important digestive enzyme secreted by the pancreas of vertebrates [23, 24]. In our results, the *rnh1* (ribonuclease/angiogenin inhibitor 1) gene (*p* value < 0.01) which inhibits *rnase1*, *rnase2* and *ang* (Angiogenin) was subjected to positive selection during the evolution of cyprinid fish. A total of 216 PSGs were identified in grass carp and blunt snout bream and then KEGG and GO enrichment analyses were performed. The function of these genes is mainly related to immune system (16 genes) (Table S8, Table S9). Besides, a total of 341 PSGs were identified in grass carp. The function of these genes is mainly related to immune system (40 genes) (Table S10, Table S11). Among these genes, 6 genes are related to the nervous system (*htr3a*, *creb5*, *nrg1*, *nrg2a*, *nrg2b*, and *nrg3*). We further used the mouse phenotype database to perform functional enrichment analysis with the grass carp PSGs (Fig. 4C). The results showed that multiple MP terms related to intestinal development were enriched, such as abnormal intestine physiology, abnormal digestive secretion, abnormal intestine morphology, abnormal intestinal epithelium morphology.

### Loss of conserved noncoding elements

**Fig. 5:**
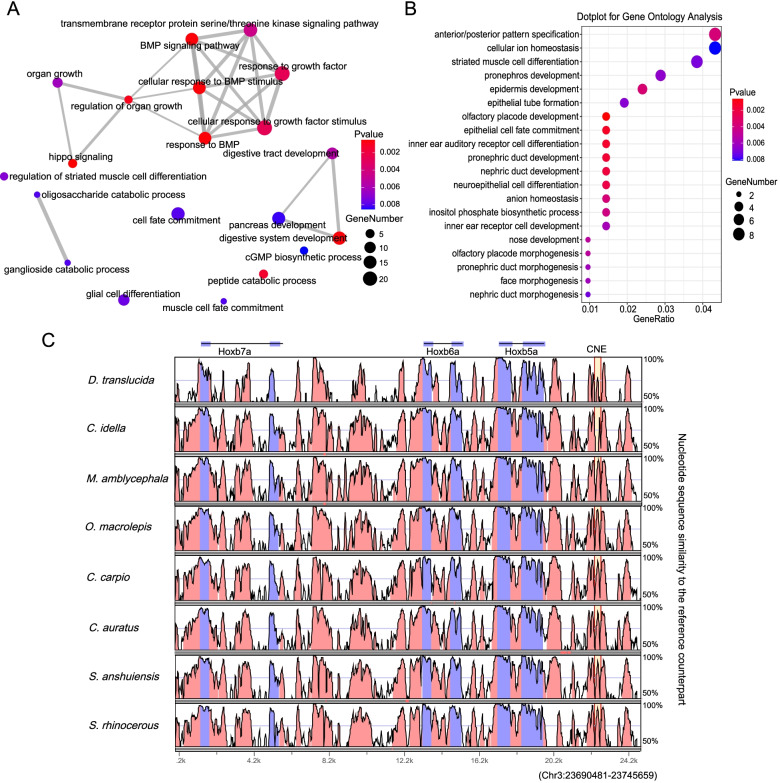
CNE adjacent gene function enrichment analysis and CNEs in HoxBa cluster of cyprinids. **A** Top 20 statistically significant (*p* value < 0.01) GO biological process terms (specific presence in zebrafish and *D. translucida*, but specific deletion in other cyprinids). **B** Top 20 statistically significant (*p* value < 0.01) GO biological process terms (specific deletion in grass carp). **C** VISTA sequence conservation plot of the grass carp specific deletion CNE around Hoxb5a, Hoxb6a and Hoxb7a, using zebrafish (GRCz11) as reference

To determine the extent of conserved noncoding element (CNE) loss in grass carp, we predicted genome-wide CNEs in zebrafish. We identified 343,313 CNEs (average size of 165.8 bp) that are conserved in zebrafish and at least one of the other nine cyprinid fishes. We searched for CNEs that are uniquely lost in each of the cyprinid fish, interestingly, *D. translucida* was found to have lost a substantially higher number of CNEs (89,346 CNEs) compared to other cyprinid fishes (Table S12). Analysis of zebrafish CNEs that are specifically present in *D. translucida* indicated that they are present in the neighbourhood of 1177 genes and these genes enriched in the functions mainly related to organ development (BMP signaling pathway, organ growth, response to growth factor and so on) and digestive system (digestive tract development, digestive system development and pancreas development) (Fig. 5A). We further analyzed zebrafish CNEs that are specifically deleted in grass carp indicated that they are present in the neighbourhood of 294 genes enriched in the functions related to olfactory placode development, olfactory placode morphogenesis, anterior/posterior pattern specification, nose development, face morphogenesis (Fig. 5B). Such as *hoxb5a*, *hoxb6a* and *hoxb7a* genes which are part of a developmental regulatory system that provides cells with specific positional identities on the anterior-posterior axis (Fig. 5C) [25, 26], and *dlx3b* and *dlx4b*, which involved in several processes, including sensory organ development, skeletal system development, and trigeminal nerve development [27, 28].

## Discussion

The grass carp has great economic value and occupies an important evolutionary position in the process of species formation. Genomic information regarding this species could help better understand the genetic mechanisms of its rapid reproductive rate as well as its environmental adaptation and unique body plan. Here, we successfully obtained 893.2 Mb chromosome-level genome assembly of the grass carp, which is much better than the assembly quality of the previously published genome [3]. The overwhelming majority of the assembled sequences (99.85%) were anchored into 24 haploid chromosomes, which is the highest among all sequenced teleost genomes. During the evolution of species, different organisms gradually formed their own unique genomes, including relatively stable DNA sequences and a fixed number of chromosomes. Almost all eukaryotes contain multiple chromosomes of varying numbers [29]. The synteny of genes over the anchored chromosomes indicated that most of the grass carp linkage groups had extensive collinearity with corresponding zebrafish chromosomes. In our results, one chromosome of grass carp only aligned to zebrafish chromosomes 10 and 22. This was consistent with previous results of grass carp, blunt snout bream, bighead carp and silver carp which were called East Asian cyprinid [3, 7, 11]. However, 25 pairs of chromosomes have been identified in zebrafish [15]. These results suggested that due to the fusion of the two ancestral chromosomes, grass carp has only 24 pairs of chromosomes, and this phenomenon exists in other East Asian cyprinids. As shown in studies on species of human and Drosophila, chromosome fusion might play important roles in adaptive evolution and speciation [30, 31], leading to species reproductive isolation and promoting the formation of new species [31, 32]. TEs are DNA sequences that can change its position within a genome, sometimes generating or reversing mutations and altering the species genetic characteristics and genome size [33]. Research has found that the proportion of TEs is positively correlated with the genome size in ruminants [20]. In cyprinid fishes, we also proved that this correlation exists.

Grass carp and blunt snout bream are typically herbivorous, a characteristic that has contributed to making them a popular breeding species. The mechanisms of feeding habit transition of grass carp and blunt snout bream and how to effectively absorb nutrients from plant to support their rapid growth are unanswered research questions [3, 6]. Grass carp and blunt snout bream are closely phylogenetically related. In the phylogenetic analysis performed at the gene level, grass carp and blunt snout bream diverged ~ 23 Mya from their common ancestor; however, the karyotypes formed a difference between the two species during the evolutionary period. Fish feeding is the result of the joint regulation of multiple organs. Fish receive information about the internal and external environment through sensory organs and convert them into neural signals, which are then transmitted to the brain. After analysis and integration in the central nervous system, the neural signals are transmitted to the effector to make the body perform corresponding physiological activities [34, 35]. Gene family analysis showed that zebrafish unique gene families are mainly related to nervous system, immune system and sensory system, implying that these functions have changed in later-formed species. A typical example is the diversity of feeding habits of species in zebrafish downstream branches. Grass carp and blunt snout bream common gene families are related to DNA recombination and antiviral immunity. Grass carp unique families have also enriched GO terms related to DNA recombination and immune system. These results suggest that DNA reorganization may play an important role in the formation of East Asian cyprinids.

The immune system is a disease defense system composed of a series of biological structures and processes in an organism. As species adapt to new environments, they are constantly exposed to new pathogens and stimuli that cause the immune system to evolve [36]. Using the resolved phylogeny of 19 teleost fishes, we identified expanded and contracted gene families, rapidly evolving gene families and positively selected genes (PSGs). GO term and KEGG pathway enrichment analysis of the PSGs and expanded gene families in the grass carp branch all exhibit enrichment in immune functions. These results indicated that immune-related genes may be beneficial for grass carp to avoid pathogens’ damage and invasion, and thus better adapt to the freshwater environment. The brain-gut axis is the biochemical signaling that occurs between the central nervous system and the gastrointestinal (GI) tract [35]. When food enters the digestive system through the mouth, it sends lots of interaction signals to the brain which contain senses, nutrition and other information [37, 38]. Interestingly, the MP terms enrichment analysis of the grass carp PSGs showed that many genes related to intestinal development. We also observed PSGs related to the nervous system development. Neuregulins (*nrgs*) are part of the EGF family and consist of four structurally related proteins. These proteins have been shown to have multiple functions in the development of the nervous system and play an important role in vertebrate embryogenesis [39, 40]. Recent research shows *nrg1* promotes intestinal stem cell proliferation and intestinal regeneration [41]. In our results, *nrg1*, *nrg2a*, *nrg2b* and *nrg3* are subject to positive selection. Besides, 7 histone gene families have expanded significantly in grass carp. Histones play a role in a variety of biological processes, such as DNA repair, gene regulation, spermatogenesis (meiosis) and chromosome condensation (mitosis) [42]. Histones mediate the orderly interaction of DNA with various regulatory elements and molecular machines by changing the open and folded state of DNA, thereby ensuring the smooth progress of various life activities [43, 44]. These results indicated that the significant expansion of histones may be related to the herbivorous traits and unique body plan of grass carp.

Vertebrate genomes contain thousands of noncoding elements selected for purification [45, 46]. Many of these conserved noncoding elements (CNEs) function as cis-regulatory elements such as enhancers, repressors and insulators [47, 48]. The evolutionary loss of CNEs plays an important role in phenotypic differences and morphological innovation [49, 50]. Except zebrafish and *D. translucida*, the other eight cyprinid fishes have larger body size and different feeding habits. In previous reports, bone morphogenetic proteins (BMPs) belong to the transforming growth factor-β (*tgf-β*) superfamily. BMPs were originally discovered for their ability to induce bone formation, and they are now known to play a vital role in all organ systems [51]. Zebrafish CNEs that are specific presence in *D. translucida* may be related to the body size and the diversity of feeding habits of cyprinid fishes. Functional enrichment analysis of these CNE associated genes indicated that mainly related to BMP-regulated growth and digestive system. We further analyzed zebrafish CNEs that are specifically lost in grass carp, the results indicated that CNE associated genes enriched in the functions related organ development and morphogenesis. These results may imply that the deletion of CNEs causes changes in gene regulatory networks and induces the diversity of cyprinids.

In previous studies, the common carp, goldfish and cavefish were heterozygous polyploids whose chromosomes were derived from allopolyploidization of different ancestors [9, 10]. The use of single-copy orthologous genes to construct a phylogenetic tree and perform positive selection analysis in this study may increase the uncertainty of the results. Therefore, we will conduct more experiments in the future to verify these results.

## Conclusions

We acquired a high-quality chromosome-level genome assembly of grass carp in the present study. The genome was 893.2 Mb in size, with a contig N50 size of 19.3 Mb, a scaffold N50 size of 35.7 Mb, and about 99.85% of the assembled contigs were anchored into 24 chromosomes. More than 95.7% of the complete BUSCO gene sequences were identified in the genome, implying the completeness of the genome. Therefore, our comprehensive evolutionary and comparative analyses have revealed numerous genetic variations related to specific traits in grass carp. Such as chromosome fusion and DNA recombination may play an important role in the formation of new species. In addition, the co-evolution of the immune system, nervous system and digestive system contributed to the herbivorous traits and unique body plan of grass carp. This study provides valuable genomic resources as well as insights into not only the understanding of the specific characteristics of grass carp but also providing data and theoretical support for subsequent research on disease resistance, molecular breeding and variety selection.

## Methods

### Sampling and sequencing

A gynogenetic grass carp was obtained from the Shanghai Ocean University (Shanghai, China). Genomic DNA was extracted from blood using a Qiagen Blood & Cell Culture DNA Mini Kit (Qiagen, Germany). Three short-insert-size libraries with an insert size of ~ 250 bp were constructed and sequenced on the BGI-seq 500 platform. A 20-kb long-read library was constructed and sequenced on the Sequel platform (Pacific Biosciences). Two Hi-C libraries were constructed with the restriction endonuclease MboI and sequenced on the BGI-seq 500 platform. RNAs from twelve different tissues, including the liver, gill, intestine, head kidney, body kidney, muscle, brain, swim bladder, spleen, blood, skin and heart were extracted using a TRIzol kit (Invitrogen, USA) and sequenced on the BGI-seq 500 platform. All the sequencing works were performed by the Beijing Genomics Institute (BGI, Shenzhen, China).

### Quality control of sequencing data

All sequencing data were filtered to reduce low-quality bases and duplicated reads using different strategies based on the platforms used. For the BGI-seq platform data (including genomic short-reads, RNA-seq reads and Hi-C reads), reads were filtered using the following steps: First, polymerase chain reaction (PCR) duplications in read pairs produced during library construction were removed. Second, adaptors were removed from the sequencing reads. Third, read pairs with more than 50% low-quality bases were removed. Fourth, read pairs were excluded if any one read had more than 10% unknown bases [20]. For the PacBio long reads, subreads were directly produced by the default parameters of the sequencing equipment (Sequel).

### De novo assembly of the grass carp genome

The Falcon software (v.1.4.2) [52] was used for the grass carp genome assembly. PacBio long reads were used to construct the skeleton of the genome. For the output of Falcon, we first used Arrow (v.2.3.3) [53] to polish the genome using long-sequencing errors; next, we applied three rounds of polishing using NGS short reads with Pilon (v.1.22) [54]. The assembly was also compared against published zebrafish mitochondrial genomes to remove mitochondrial sequences. To generate the linear chromosome-scale genome assembly of grass carp, the Hi-C data were mapped to these segments using BWA (v.0.7.17-r1188) software [55]. Any two segments which showed inconsistent connection with information from the raw scaffold were checked manually. These corrected scaffolds were then assembled with LACHESIS [56]. To assess the quality of assembly, Hi-C data were mapped to chromosomes using HiC-Pro software (v.2.11.1) [57] and placement and orientation errors exhibiting obvious discrete chromatin interaction patterns were manually adjusted. The interaction matrix of each chromosome was visualized with heatmaps at the 500 kb resolution.

### Heterozygosity analysis

BGI-seq platform short reads were mapped to the assembled genome with Bowtie2 [58]. Regions with alignment gaps were realigned with GATK (v.4.1.2.0) [59] and duplicate reads marked with Picard Tools (http://picard.sourceforge.net). Sequence variations were called with GATK.

### Repetitive element annotation

Transposable elements (TEs) in teleost genomes were detected by combining homology-based and de novo predictions. For homology-based detection, RepeatMasker and RepeatProteinMask were used to screen the teleost fish genomes for known transposable elements against the RepBase library (http://www.repeatmasker.org/). De novo TEs in the genome were identified by RepeatMasker based on a de novo repeat library constructed by RepeatModeller and LTR_FINDER (v.1.0.5) [60]. Tandem repeats were detected using the program Tandem Repeats Finder (TRF, v.4.07b) [61] with default parameters.

### Genome annotation and functional annotation

Gene prediction and functional annotation were performed through a combination of homology-based prediction, de novo prediction and transcriptome-based prediction methods. Protein sequences from *C. carpio*, *Sinocyclocheilus anshuiensis*, *Danio rerio* and *M. amblycephala* were aligned to the grass carp genome using TblastN (E-value ≤1e-5). The BLAST hits were conjoined by GeneWise (v2.4.1) [62] for accurate spliced alignments. For de novo prediction, three tools, Augustus (v.2.7) [63], GlimmerHMM (v.3.02) [64] and SNAP (version 2006-07-28) [65], were used to predict the genes in the repeat-masked genome sequences. The RNA-seq reads from twelve tissues were mapped onto the genome assembly using TopHat (v.2.1.1) [66], and then Cufflinks (v2.2.1) [67] was used to assemble the transcripts into gene models. Gene predictions from the de novo approach, homology-based approach and RNA-seq-based evidence were merged to form a comprehensive consensus gene set using the software EVM [68]. To calculate gene expression for 12 tissues, Hisat2 (v.2.1.0) [69] was used for multiple sequence alignment of clean data, and HTSeq (v.0.11.2) [70] was used to calculate TPM value for gene expression. Although the whole genomic sequence of *M. amblycephala* has been published, the resulting document of gene structure prediction has not been made public [7, 71]. Therefore, the same methods were used to make gene structure prediction, and we re-obtained the genomic structure information and gene sequence. Subsequently, the newly obtained protein sequence was compared with the published sequence using Busco [72]. The complete Benchmarking Universal Single-Copy Orthologs (BUSCOs) in the newly predicted and published sequences were 92.4 and 92.89%, respectively (Table S3). And the missing BUSCOs in the newly predicted and published genome were 5.0 and 5.67%, respectively [7]. These results indicate that our prediction is similar to previous report. To achieve the functional annotation, the predicted protein sequences were aligned against public databases, including NCBI non-redundant (NR), Kyoto Encyclopedia of Genes and Genomes (KEGG) [73] and Mammalian Phenotypes (MP) (http://www.mousemine.org) with BLASTP (E-value ≤1e-10). Additionally, protein motifs and domains were annotated by searching the InterPro and Gene Ontology (GO) databases using InterProScan (v.4.8) [74].

### Identification of orthologous genes

Orthologs were identified in the assembled genomes of 19 sequenced species (Table S1), along with the species with published genome sequences using the OrthoMCL pipeline (v.2.0.9) [75]. Briefly, the protein-coding genes of the published species were downloaded from the NCBI database except grass carp and blunt snout bream. To improve the accuracy of the analysis, genes that encode shorter than 30 amino acids or have early stop codons in the coding regions were removed. All the remaining genes were aligned and reciprocally compared, and the reciprocal best similarity pairs among species were considered as putative orthologs after further evaluation using MCscan software [76].

### Phylogenetic tree construction and divergence time evaluation

All the 5067 single-copy homologous genes identified among 19 teleost fishes were aligned and concatenated into supergenes for phylogenetic relationship analyses. Maximum likelihood-based phylogenetic analysis was conducted using RAxML (v.8.2.12) [77]. Meanwhile, species trees were also constructed using MP-EST (v.2.0) [18] and ASTRAL [17]. Divergence times of these species were then estimated on the basis of the 4dTV sequences via Bayesian relaxed molecular clock approach using MCMCtree program in the PAML package (v.4.8) [78]. Fossil records downloaded from the TIMETREE website (http://www.timetree.org) were used for calibrating our calculated divergence time.

### Conserved non-exonic element (CNE) annotation

We obtained the synteny blocks of 10 cyprinid fishes by aligning to the zebrafish genome (GRCz11) using LAST (version 1066) [79]. Each genome was aligned to the zebrafish genome using the “lastal” command with the parameter: -E 0.05. Then, we used the “maf-swap” command to change the order of the sequences in MAF-format alignments, and obtained the best pairwise aligned blocks. Four-fold degenerate (4D) sites of zebrafish genes were extracted from the multiple alignments. These 4D sites were used to build a neutral model using PhyloFit in the PHAST (v.1.4) package [80] (general reversible “REV” substitution model). PhastCons was then run with rho-estimation mode on each of the zebrafish chromosomal alignments to obtain a conserved model for each chromosome. These conserved models were averaged into one model using PhyloBoot. Subsequently, conserved elements were predicted in the multiple alignments using PhastCons with the following inputs and parameters: the neutral and conserved models, target coverage of input alignments = 0.3 and average length of conserved sequence = 45 bp. To assess the sensitivity of this approach in identifying functional elements, the PhastCons elements were compared against zebrafish protein-coding genes. The conserved elements were classified as follows. Firstly, elements that were shorter than 30 bp were excluded. Secondly, the conserved elements were segregated into repetitive sequences (≥ 30% of bases were repeat-masked), exonic (overlapping known protein-coding genes or noncoding RNA in zebrafish gene-build), and non-exonic. Thirdly, the non-exonic elements were filtered against zebrafish mRNAs (~ 31,000), spliced ESTs (~ 1.5 million) downloaded from the UCSC Genome Browser for ‘danRer11’ assembly, and vertebrate proteins obtained from Uniprot (~ 240,000 proteins in ‘complete’ and ‘reference’ vertebrate proteomes; BLASTX at 1e-5). The conserved elements that were neither exonic based on known genes, nor potentially protein-coding, nor repetitive sequence were classified as CNEs.

### Conservation or loss of CNEs in teleost fish genomes

A CNE was considered present in a cyprinid fish genome if it showed a coverage of at least 30% with a zebrafish CNE in Multiz [81] alignment. To identify CNEs that could have been missed in the Multiz alignments due to rearrangements in the genomes, or due to partitioning of the CNEs among cyprinid fish duplicate genes, we searched the zebrafish CNEs against the genome of the cyprinid fish using BLASTN (E-value <1e-10; ≥ 80% identity; ≥ 30% coverage). Those CNEs that had no significant match in a cyprinid fish genome were considered as missing in that genome. The method of CNEs annotation refers to previous research [20, 82]. We visualized CNEs using the online tool VISTA (https://genome.lbl.gov/vista) [83].

### Expansion and contraction of gene families

For greater insight into the evolutionary dynamics of the genes, the expansion and contraction of the gene ortholog clusters were determined (*p* value < 0.01) among the 19 species by comparing cluster sizes between ancestors and each current species using CAFÉ software (v.4.2.1) [84]. The gene gain and loss along each lineage of the RAxML tree were calculated by CAFÉ software with a random birth and death process model. A probabilistic graphical model (PGM) was introduced to calculate the probability of transitions in gene family size from parent to child on the phylogeny. The expanded and contracted gene families in grass carp were identified by comparison with other species, and expanded and contracted gene families in other species were identified by comparison with ancestors. KEGG and GO analyses were conducted based on gene families exclusively presented and specifically expanded and contracted in cyprinid fish using clusterProfiler [85].

### Identification of positively selected genes (PSGs)

All one-to-one orthologous genes extracted from 19 species were used to identify PSGs. The multiple sequence alignments were generated and used to estimate three types of ω (the ratio of the rate of nonsynonymous substitutions to the rate of synonymous substitutions) using branch model in the codeml program of the PAML package (v.4.8) [78]. Branch model (model = 2, NS sites = 0) was used to detect ω of appointed branch to test (ω0) and average ω of all the other branches (ω1) and the mean of whole branches (ω2). Then χ2 test was used to check whether ω0 was significantly higher than ω1 and ω2 under the threshold *p* value < 0.01, which hinted that these genes would be under positive selection or fast evolution.

## Supplementary Information

**Additional file 1: Figure S1**. Distribution density of genes on grass carp chromosomes. Color from blue to red indicates increased gene density.

**Additional file 2: Figure S2**. Ratio of syntenic depth between zebrafish and grass carp. Syntenic blocks of zebrafish per grass carp gene (left) and syntenic blocks of grass carp per zebrafish gene (right) are shown indicating 2:1 pattern of zebrafish to grass carp.

**Additional file 3: Figure S3**. Coalescent species tree inferred by ASTRAL and MP-EST. Five thousand sixty-seven protein coding gene trees were used to infer the species tree using (A) ASTRAL and (B) MP-EST.

**Additional file 4: Figure S4**. KEGG pathway annotation of grass carp newly evolved genes.

**Additional file 5: Table S1**. Genomic information statistics of 19 teleosts (genome size information from NCBI public database).

**Additional file 6: Table S2**. The summary of previous and current grass carp genomes.

**Additional file 7: Table S3**. Completeness assessment of grass carp (previous and current genomes) and blunt snout bream (newly predicted genes) genomes by BUSCO.

**Additional file 8: Table S4**. The statistically significant (*p* value < 0.001) GO biological process terms of grass carp and blunt snout bream common gene families.

**Additional file 9: Table S5**. The top 20 statistically significant KEGG pathways of grass carp and blunt snout bream specially expanded gene family.

**Additional file 10: Table S6**. The top 20 statistically significant GO biological process terms of grass carp specifically expanded gene families.

**Additional file 11: Table S7**. The top 20 statistically significant KEGG pathways of grass carp specifically expanded gene families.

**Additional file 12: Table S8**. The top 20 statistically significant GO biological process terms of grass carp and blunt snout bream PSGs.

**Additional file 13: Table S9**. The top 20 statistically significant KEGG pathways of grass carp and blunt snout bream PSGs.

**Additional file 14: Table S10**. The top 20 statistically significant GO biological process terms of grass carp PSGs.

**Additional file 15: Table S11**. The top 20 statistically significant KEGG pathways of grass carp PSGs.

**Additional file 16: Table S12**. Number of CNEs specific presence and deletion in one of the cyprinid genomes.

## Data Availability

The raw genome, transcriptome and Hi-C data have been deposited in the SRA under bioproject number PRJNA745278 (https://www.ncbi.nlm.nih.gov/bioproject/PRJNA745278). The final chromosome assembly was submitted to NCBI under bioproject number PRJNA745929 (https://www.ncbi.nlm.nih.gov/bioproject/PRJNA745929).
