# Supplementary figures and images for: Chromosome-level genome assembly of grass carp (Ctenopharyngodon idella) provides insights into its genome evolution
Source: BMC Genomics. 2022 Apr 7;23:271. doi: 10.1186/s12864-022-08503-x (PMC8988418; doi:10.1186/s12864-022-08503-x)

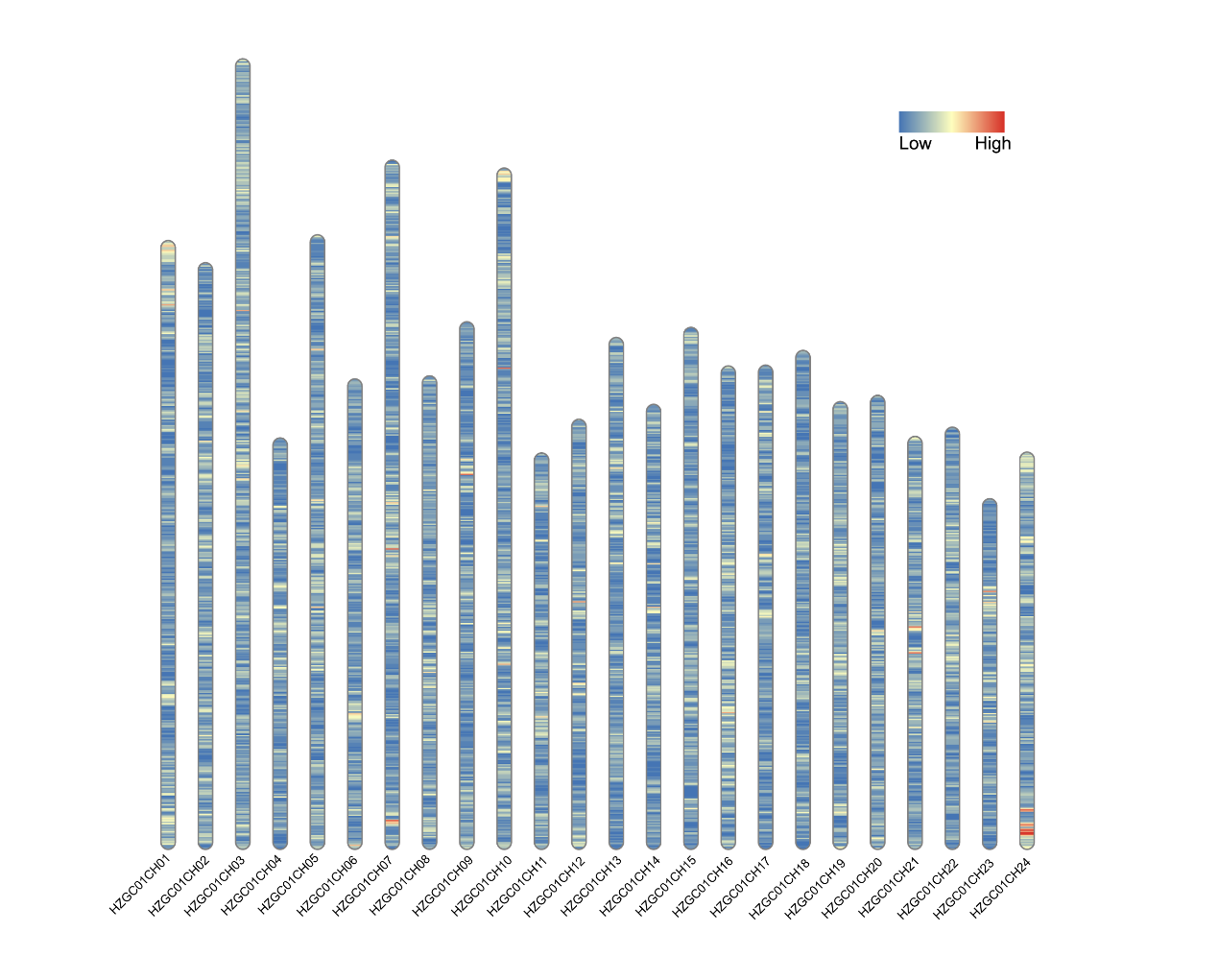

Supplement: Supplementary file 1 — Additional file 1: Figure S1. Distribution density of genes on grass carp chromosomes. Color from blue to red indicates increased gene density. [file 12864_2022_8503_MOESM1_ESM.docx]

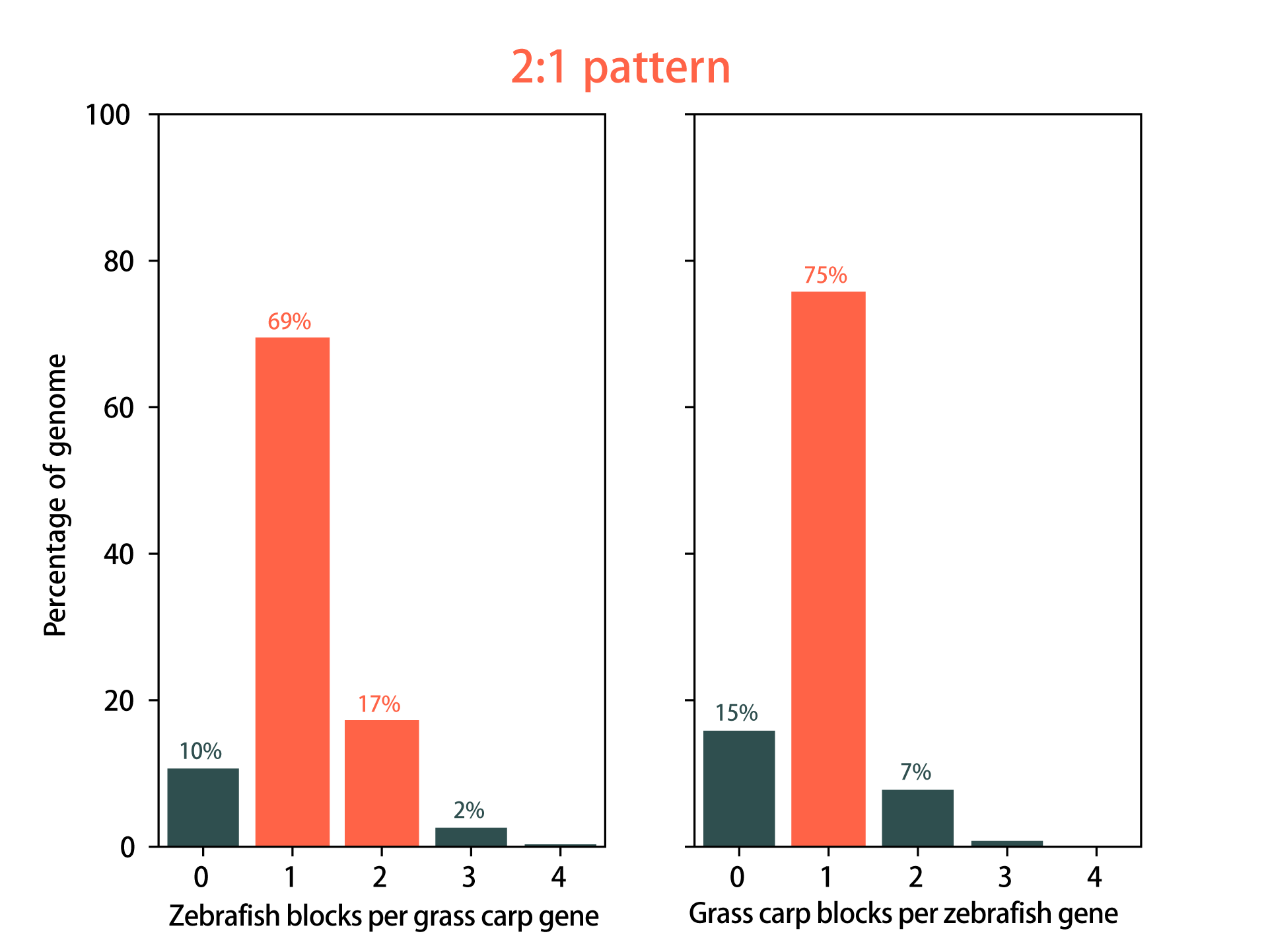

Supplement: Supplementary file 2 — Additional file 2: Figure S2. Ratio of syntenic depth between zebrafish and grass carp. Syntenic blocks of zebrafish per grass carp gene (left) and syntenic blocks of grass carp per zebrafish gene (right) are shown indicating 2:1 pattern of zebrafish to grass carp. [file 12864_2022_8503_MOESM2_ESM.docx]

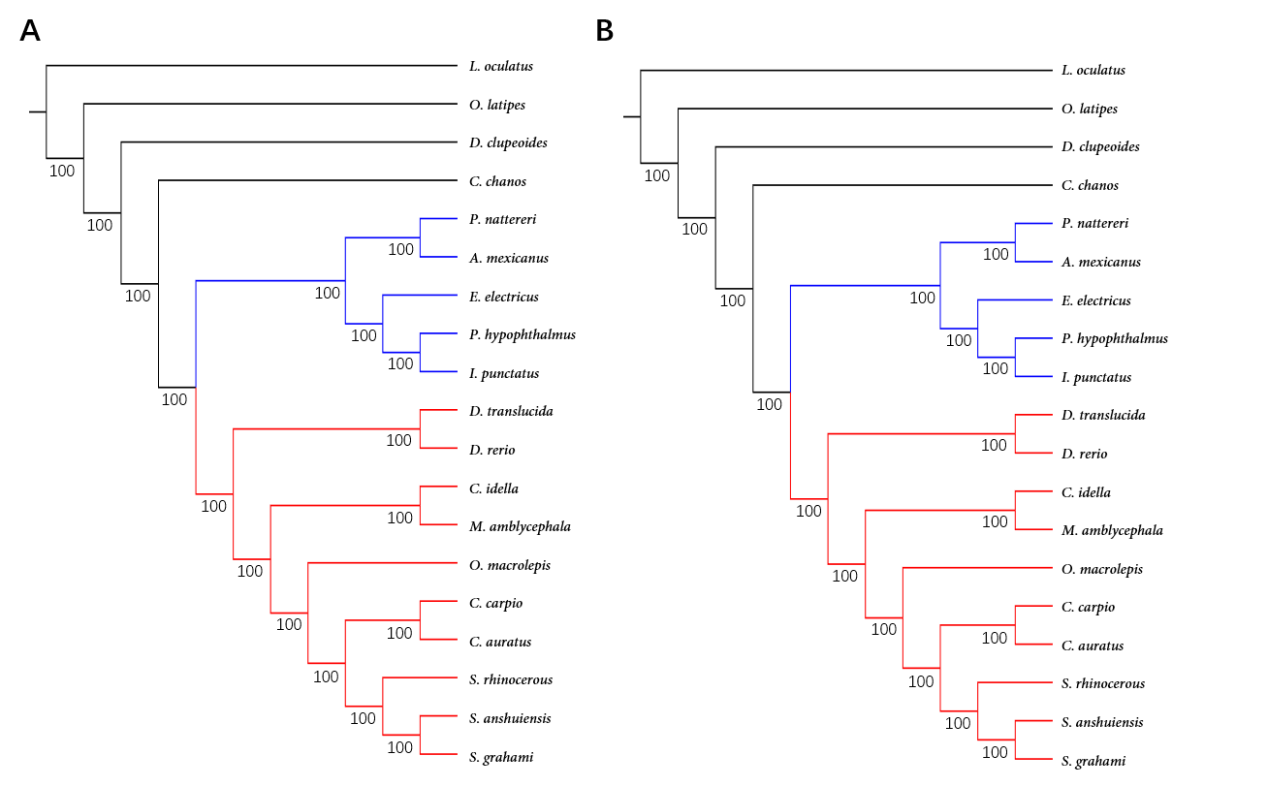

Supplement: Supplementary file 3 — Additional file 3: Figure S3. Coalescent species tree inferred by ASTRAL and MP-EST. Five thousand sixty-seven protein coding gene trees were used to infer the species tree using (A) ASTRAL and (B) MP-EST. [file 12864_2022_8503_MOESM3_ESM.docx]

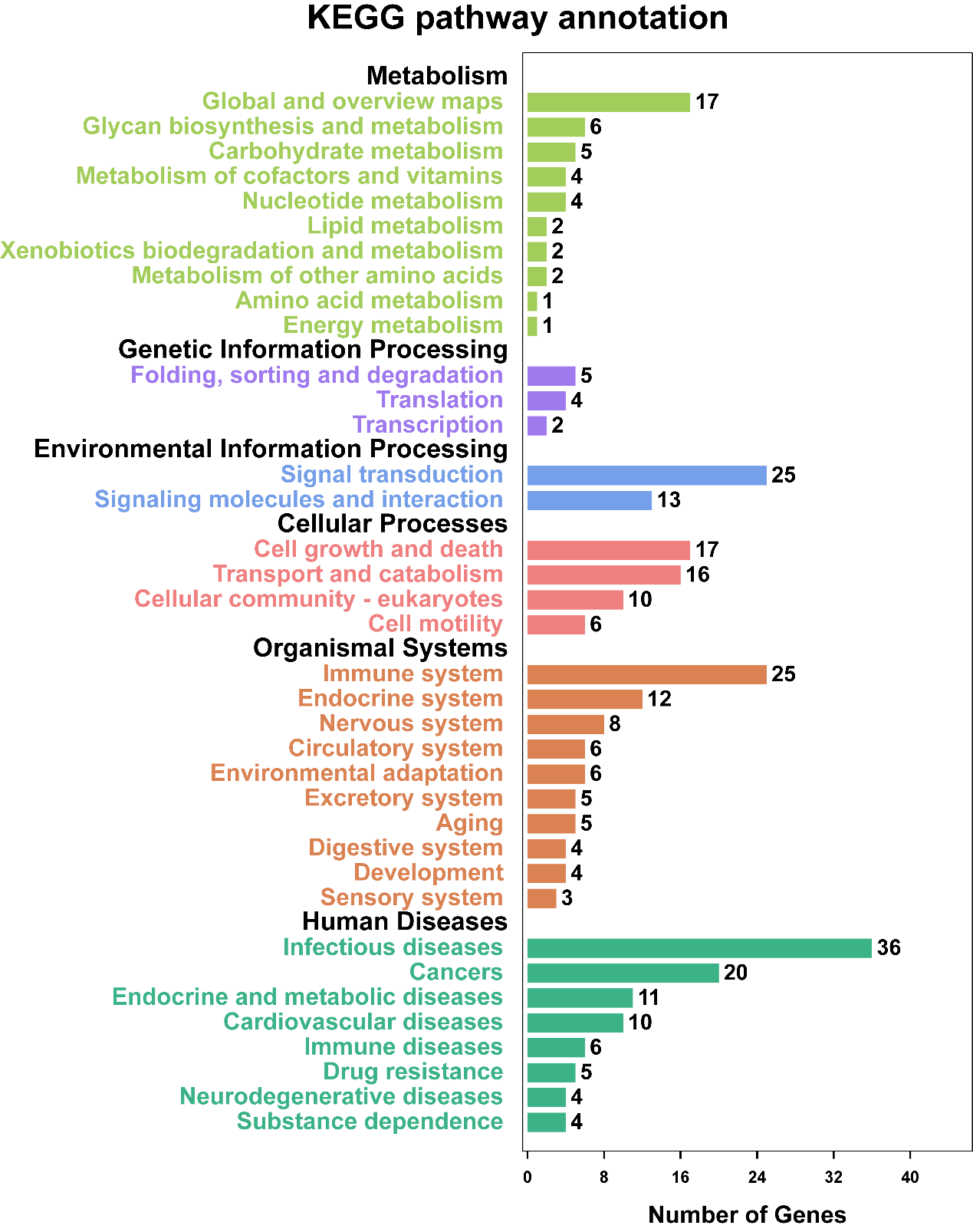

Supplement: Supplementary file 4 — Additional file 4: Figure S4. KEGG pathway annotation of grass carp newly evolved genes. [file 12864_2022_8503_MOESM4_ESM.docx]
