# Supplementary material for: Chromosome-level genome assembly of grass carp (Ctenopharyngodon idella) provides insights into its genome evolution
Source: BMC Genomics. 2022 Apr 7;23:271. doi: 10.1186/s12864-022-08503-x (PMC8988418; doi:10.1186/s12864-022-08503-x)
Supplement: Supplementary file 5 — Additional file 5: Table S1. Genomic information statistics of 19 teleosts (genome size information from NCBI public database). [file 12864_2022_8503_MOESM5_ESM.docx]

| Family | Scientific name | Genome size (Mb) | GC (%) | Scaffold N50 (Mb) | Repeat rates (%) | Complete busco (%) |
| --- | --- | --- | --- | --- | --- | --- |
| Cypriniformes | *Danio rerio* | 1679.20 | 36.60 | 52.19 | 54.47 | 98.7 |
| Cypriniformes | *Onychostoma macrolepis* | 883.23 | 37.23 | 34.07 | 40.67 | 93.3 |
| Cypriniformes | *Carassius auratus* | 1820.41 | 37.48 | 22.76 | 34.12 | 98.9 |
| Cypriniformes | *Cyprinus carpio* | 1670.38 | 37.00 | 7.83 | 33.41 | 83.5 |
| Cypriniformes | *Danionella translucida* | 725.70 | 38.57 | 0.34 | 33.34 | 84.9 |
| Cypriniformes | *Sinocyclocheilus grahami* | 1567.44 | 37.54 | 1.16 | 37.46 | 94.7 |
| Cypriniformes | *Sinocyclocheilus rhinocerous* | 1521.48 | 37.18 | 0.95 | 34.64 | 97.3 |
| Cypriniformes | *Sinocyclocheilus anshuiensis* | 1512.90 | 37.28 | 1.28 | 36.16 | 98.1 |
| Cypriniformes | *Ctenopharyngodon idella* | 893.05 | 37.48 | 35.66 | 43.26 | 95.7 |
| Cypriniformes | *Megalobrama amblycephala* | 1073.96 | 37.40 | 1.40 | 45.68 | 92.4 |
| Characiformes | *Astyanax mexicanus* | 1291.60 | 38.00 | 35.38 | 42.67 | 96.9 |
| Characiformes | *Pygocentrus nattereri* | 1252.07 | 40.45 | 1.44 | 40.59 | 98.0 |
| Gymnotiformes | *Electrophorus electricus* | 534.84 | 42.56 | 0.61 | 14.94 | 96.8 |
| Siluriformes | *Pangasianodon hypophthalmus* | 758.80 | 38.86 | 26.46 | 30.74 | 97.4 |
| Siluriformes | *Ictalurus punctatus* | 771.90 | 39.70 | 27.42 | 34.47 | 97.2 |
| Gonorynchiformes | *Chanos chanos* | 652.70 | 41.53 | 50.30 | 16.18 | 98.9 |
| Clupeiformes | *Denticeps clupeoides* | 562.75 | 43.69 | 22.79 | 22.02 | 97.5 |
| Beloniformes | *Oryzias latipes* | 733.57 | 40.84 | 31.22 | 32.65 | 98.1 |
| Semionotiformes | *Lepisosteus oculatus* | 869.42 | 39.59 | 50.34 | 18.47 | 94.2 |
