# Supplementary material for: Chromosome-level genome assembly of grass carp (Ctenopharyngodon idella) provides insights into its genome evolution
Source: BMC Genomics. 2022 Apr 7;23:271. doi: 10.1186/s12864-022-08503-x (PMC8988418; doi:10.1186/s12864-022-08503-x)
Supplement: Supplementary file 6 — Additional file 6: Table S2. The summary of previous and current grass carp genomes. [file 12864_2022_8503_MOESM6_ESM.docx]

|  | Previous genome | Current genome |
| --- | --- | --- |
| **Scaffold statistics** |  |  |
| Scaffold number | 164368 | 30 |
| Minimum length (bp) | 200 | 7653 |
| Scaffold length (bp) | 900506596 | 893198495 |
| Scaffold N50 (bp) | 6428943 | 35662356 |
| Scaffold N90 (bp) | 179214 | 30301089 |
| Scaffold maximum (bp) | 19571558 | 58213323 |
| **Contig statistics** |  |  |
| Contig number | 199265 | 180 |
| Contig length (bp) | 865436146 | 893048495 |
| Contig N50 (bp) | 39122 | 19319444 |
| Contig N90 (bp) | 4332 | 3248144 |
| GC (%) | 37.42 | 37.48 |
