# Supplementary material for: Chromosome-level genome assembly of grass carp (Ctenopharyngodon idella) provides insights into its genome evolution
Source: BMC Genomics. 2022 Apr 7;23:271. doi: 10.1186/s12864-022-08503-x (PMC8988418; doi:10.1186/s12864-022-08503-x)
Supplement: Supplementary file 7 — Additional file 7: Table S3. Completeness assessment of grass carp (previous and current genomes) and blunt snout bream (newly predicted genes) genomes by BUSCO. [file 12864_2022_8503_MOESM7_ESM.docx]

| Actinopterygii_odb9 | Grass carp (previous) | Grass carp (current) | Blunt snout bream |
| --- | --- | --- | --- |
| Complete BUSCOs (C) | 3835 | 4386 | 4233 |
| Complete and single-copy BUSCOs (S) | 3600 | 4050 | 3978 |
| Complete and duplicated BUSCOs (D) | 235 | 336 | 255 |
| Fragmented BUSCOs (F) | 498 | 114 | 119 |
| Missing BUSCOs (M) | 251 | 84 | 232 |
| Total BUSCO groups searched | 4584 | 4584 | 4584 |
| Complete BUSCOs (%) | 83.6 | 95.7 | 92.4 |
