# Supplementary material for: Chromosome-level genome assembly of grass carp (Ctenopharyngodon idella) provides insights into its genome evolution
Source: BMC Genomics. 2022 Apr 7;23:271. doi: 10.1186/s12864-022-08503-x (PMC8988418; doi:10.1186/s12864-022-08503-x)
Supplement: Supplementary file 8 — Additional file 8: Table S4. The statistically significant (p value < 0.001) GO biological process terms of grass carp and blunt snout bream common gene families. [file 12864_2022_8503_MOESM8_ESM.docx]

| GO ID | Namespace | Description | Count | *p* value |
| --- | --- | --- | --- | --- |
| GO:0006310 | Biological process | DNA recombination | 14 | 7.29e-13 |
| GO:0015074 | Biological process | DNA integration | 16 | 1.42e-10 |
| GO:0006313 | Biological process | Transposition, DNA-mediated | 9 | 7.35e-09 |
| GO:0044826 | Biological process | Viral genome integration into host DNA | 6 | 2.17e-06 |
| GO:0003823 | Molecular function | Antigen binding | 4 | 8.43e-05 |
| GO:0032197 | Biological process | Transposition, RNA-mediated | 6 | 9.05e-05 |
| GO:0034765 | Biological process | Regulation of ion transmembrane transport | 11 | 0.000165 |
| GO:0019087 | Biological process | Transformation of host cell by virus | 3 | 0.000377 |
| GO:0019068 | Biological process | Virion assembly | 5 | 0.000436 |
| GO:0044179 | Biological process | Hemolysis in other organism | 6 | 0.000594 |
| GO:0010498 | Biological process | Proteasomal protein catabolic process | 2 | 0.001180 |
| GO:0071625 | Biological process | Vocalization behavior | 4 | 0.001289 |
