# Supplementary material for: Chromosome-level genome assembly of grass carp (Ctenopharyngodon idella) provides insights into its genome evolution
Source: BMC Genomics. 2022 Apr 7;23:271. doi: 10.1186/s12864-022-08503-x (PMC8988418; doi:10.1186/s12864-022-08503-x)
Supplement: Supplementary file 9 — Additional file 9: Table S5. The top 20 statistically significant KEGG pathways of grass carp and blunt snout bream specially expanded gene family. [file 12864_2022_8503_MOESM9_ESM.docx]

| Pathway ID | KEGG class | Pathway | Count | *p* value |
| --- | --- | --- | --- | --- |
| ko05322 | Immune diseases | Systemic lupus erythematosus | 9 | 1.98e-10 |
| ko04514 | Signaling molecules and interaction | Cell adhesion molecules (CAMs) | 9 | 1.17e-06 |
| ko05310 | Immune diseases | Asthma | 4 | 1.15e-05 |
| ko04672 | Immune system | Intestinal immune network for IgA production | 5 | 2.44e-05 |
| ko05150 | Infectious diseases | Staphylococcus aureus infection | 5 | 2.63e-05 |
| ko05140 | Infectious diseases | Leishmaniasis | 5 | 5.17e-05 |
| ko04640 | Immune system | Hematopoietic cell lineage | 5 | 7.00e-05 |
| ko05332 | Immune diseases | Graft-versus-host disease | 4 | 0.000128 |
| ko04145 | Transport and catabolism | Phagosome | 6 | 0.000153 |
| ko05330 | Immune diseases | Allograft rejection | 4 | 0.000187 |
| ko05320 | Immune diseases | Autoimmune thyroid disease | 4 | 0.000264 |
| ko04940 | Endocrine and metabolic diseases | Type I diabetes mellitus | 4 | 0.000384 |
| ko05152 | Infectious diseases | Tuberculosis | 6 | 0.000426 |
| ko05321 | Immune diseases | Inflammatiory bowel disease (IBD) | 4 | 0.000431 |
| ko04612 | Immune system | Antigen processing and presentation | 4 | 0.000886 |
| ko05416 | Cardiovascular diseases | Viral myocarditis | 4 | 0.001016 |
| ko05323 | Immune diseases | Rheumatoid arthritis | 4 | 0.001427 |
| ko04658 | Immune system | Th1 and Th2 cell differentiation | 4 | 0.002403 |
| ko05164 | Infectious diseases | Influenza A | 5 | 0.002416 |
| ko05168 | Infectious diseases | Herpes simplex infection | 5 | 0.003922 |
