# Supplementary material for: Chromosome-level genome assembly of grass carp (Ctenopharyngodon idella) provides insights into its genome evolution
Source: BMC Genomics. 2022 Apr 7;23:271. doi: 10.1186/s12864-022-08503-x (PMC8988418; doi:10.1186/s12864-022-08503-x)
Supplement: Supplementary file 10 — Additional file 10: Table S6. The top 20 statistically significant GO biological process terms of grass carp specifically expanded gene families. [file 12864_2022_8503_MOESM10_ESM.docx]

| GO ID | Description | Count | *p* value |
| --- | --- | --- | --- |
| GO:0006955 | Immune response | 25 | 3.41e-10 |
| GO:0002252 | Immune effector process | 15 | 1.18e-08 |
| GO:0006952 | Defense response | 19 | 4.79e-08 |
| GO:0002376 | Immune system process | 28 | 8.99e-07 |
| GO:0009615 | Response to virus | 9 | 3.24e-06 |
| GO:0006968 | Cellular defense response | 5 | 4.20e-06 |
| GO:0009605 | Response to external stimulus | 26 | 6.94e-06 |
| GO:0006950 | Response to stress | 34 | 1.73e-05 |
| GO:0051707 | Response to other organism | 13 | 1.74e-05 |
| GO:0043207 | Response to external biotic stimulus | 13 | 1.82e-05 |
| GO:0070936 | Protein K48-linked ubiquitination | 5 | 2.03e-05 |
| GO:0009607 | Response to biotic stimulus | 13 | 2.34e-05 |
| GO:0050896 | Response to stimulus | 74 | 5.82e-05 |
| GO:0002250 | Adaptive immune response | 7 | 6.96e-05 |
| GO:0015074 | DNA integration | 7 | 7.54e-05 |
| GO:0022610 | Biological adhesion | 19 | 9.18e-05 |
| GO:0042110 | T cell activation | 8 | 9.59e-05 |
| GO:0070489 | T cell aggregation | 8 | 9.59e-05 |
| GO:0071593 | Lymphocyte aggregation | 8 | 0.000102 |
| GO:0070486 | Leukocyte aggregation | 8 | 0.000115 |
