# Supplementary material for: Chromosome-level genome assembly of grass carp (Ctenopharyngodon idella) provides insights into its genome evolution
Source: BMC Genomics. 2022 Apr 7;23:271. doi: 10.1186/s12864-022-08503-x (PMC8988418; doi:10.1186/s12864-022-08503-x)
Supplement: Supplementary file 11 — Additional file 11: Table S7. The top 20 statistically significant KEGG pathways of grass carp specifically expanded gene families. [file 12864_2022_8503_MOESM11_ESM.docx]

| Pathway ID | KEGG class | Pathway | Count | *p* value |
| --- | --- | --- | --- | --- |
| ko04514 | Signaling molecules and interaction | Cell adhesion molecules (CAMs) | 19 | 1.95e-12 |
| ko05322 | Immune diseases | Systemic lupus erythematosus | 12 | 7.12e-11 |
| ko04612 | Immune system | Antigen processing and presentation | 10 | 2.75e-08 |
| ko04640 | Immune system | Hematopoietic cell lineage | 9 | 4.00e-07 |
| ko05332 | Immune diseases | Graft-versus-host disease | 7 | 1.38e-06 |
| ko05416 | Cardiovascular diseases | Viral myocarditis | 8 | 5.44e-06 |
| ko04672 | Immune system | Intestinal immune network for IgA production | 7 | 1.06e-05 |
| ko04145 | Transport and catabolism | Phagosome | 10 | 1.12e-05 |
| ko05150 | Infectious diseases | Staphylococcus aureus infection | 7 | 1.17e-05 |
| ko05330 | Immune diseases | Allograft rejection | 6 | 3.60e-05 |
| ko05320 | Immune diseases | Autoimmune thyroid disease | 6 | 5.99e-05 |
| ko05168 | Infectious diseases | Herpes simplex infection | 10 | 9.74e-05 |
| ko04940 | Endocrine and metabolic diseases | Type I diabetes mellitus | 6 | 0.000104 |
| ko05310 | Immune diseases | Asthma | 4 | 0.000252 |
| ko00040 | Carbohydrate metabolism | Pentose and glucuronate interconversions | 4 | 0.000383 |
| ko05034 | Substance dependence | Alcoholism | 8 | 0.000497 |
| ko04015 | Signal transduction | Rap1 signaling pathway | 10 | 0.000662 |
| ko05323 | Immune diseases | Rheumatoid arthritis | 6 | 0.000696 |
| ko04650 | Immune system | Natural killer cell mediated cytotoxicity | 6 | 0.001197 |
| ko00980 | Xenobiotics biodegradation and metabolism | Metabolism of xenobiotics by cytochrome P450 | 4 | 0.001407 |
