# Supplementary material for: Chromosome-level genome assembly of grass carp (Ctenopharyngodon idella) provides insights into its genome evolution
Source: BMC Genomics. 2022 Apr 7;23:271. doi: 10.1186/s12864-022-08503-x (PMC8988418; doi:10.1186/s12864-022-08503-x)
Supplement: Supplementary file 12 — Additional file 12: Table S8. The top 20 statistically significant GO biological process terms of grass carp and blunt snout bream PSGs. [file 12864_2022_8503_MOESM12_ESM.docx]

| GO ID | Description | Count | *p* value |
| --- | --- | --- | --- |
| GO:0006955 | Immune response | 15 | 3.75e-09 |
| GO:0006968 | Cellular defense response | 5 | 2.35e-07 |
| GO:0002376 | Immune system process | 16 | 5.54e-07 |
| GO:0006952 | Defense response | 9 | 4.29e-05 |
| GO:0042742 | Defense response to bacterium | 4 | 7.65e-05 |
| GO:0050896 | Response to stimulus | 32 | 0.000108 |
| GO:0002757 | Immune response-activating signal transduction | 4 | 0.000425 |
| GO:0002764 | Immune response-regulating signaling pathway | 4 | 0.000644 |
| GO:0050851 | Antigen receptor-mediated signaling pathway | 3 | 0.000662 |
| GO:0050853 | B cell receptor signaling pathway | 2 | 0.000878 |
| GO:0002429 | Immune response-activating cell surface receptor signaling pathway | 3 | 0.000949 |
| GO:0002253 | Activation of immune response | 4 | 0.000993 |
| GO:0002768 | Immune response-regulating cell surface receptor signaling pathway | 3 | 0.001657 |
| GO:0050830 | Defense response to Gram-positive bacterium | 2 | 0.001788 |
| GO:0050829 | Defense response to Gram-negative bacterium | 2 | 0.002007 |
| GO:0050778 | Positive regulation of immune response | 4 | 0.002385 |
| GO:0001833 | Inner cell mass cell proliferation | 1 | 0.003727 |
| GO:0043152 | Induction of bacterial agglutination | 1 | 0.003727 |
| GO:0044245 | Polysaccharide digestion | 1 | 0.003727 |
| GO:0050828 | Regulation of liquid surface tension | 1 | 0.003727 |
