# Supplementary material for: Chromosome-level genome assembly of grass carp (Ctenopharyngodon idella) provides insights into its genome evolution
Source: BMC Genomics. 2022 Apr 7;23:271. doi: 10.1186/s12864-022-08503-x (PMC8988418; doi:10.1186/s12864-022-08503-x)
Supplement: Supplementary file 13 — Additional file 13: Table S9. The top 20 statistically significant KEGG pathways of grass carp and blunt snout bream PSGs. [file 12864_2022_8503_MOESM13_ESM.docx]

| Pathway ID | KEGG class | Pathway | Count | *p* value |
| --- | --- | --- | --- | --- |
| ko05332 | Immune diseases | Graft-versus-host disease | 5 | 1.03e-05 |
| ko04650 | Immune system | Natural killer cell mediated cytotoxicity | 6 | 1.06e-05 |
| ko04668 | Signal transduction | TNF signaling pathway | 5 | 0.000253 |
| ko04940 | Endocrine and metabolic diseases | Type I diabetes mellitus | 4 | 0.000427 |
| ko04060 | Signaling molecules and interaction | Cytokine-cytokine receptor interaction | 6 | 0.000528 |
| ko04514 | Signaling molecules and interaction | Cell adhesion molecules (CAMs) | 6 | 0.00064 |
| ko05143 | Infectious diseases | African trypanosomiasis | 3 | 0.000716 |
| ko04064 | Signal transduction | NF-kappa B signaling pathway | 4 | 0.001419 |
| ko05330 | Immune diseases | Allograft rejection | 3 | 0.003546 |
| ko04142 | Transport and catabolism | Lysosome | 4 | 0.004067 |
| ko05320 | Immune diseases | Autoimmune thyroid disease | 3 | 0.004207 |
| ko04130 | Folding, sorting and degradation | SNARE interactions in vesicular transport | 2 | 0.009358 |
| ko05323 | Immune diseases | Rheumatoid arthritis | 3 | 0.010425 |
| ko04623 | Immune system | Cytosolic DNA-sensing pathway | 2 | 0.01963 |
| ko05162 | Infectious diseases | Measles | 3 | 0.022316 |
| ko05144 | Infectious diseases | Malaria | 2 | 0.026694 |
| ko05163 | Infectious diseases | Human cytomegalovirus infection | 4 | 0.035883 |
| ko04210 | Cell growth and death | Apoptosis | 3 | 0.045119 |
| ko04672 | Immune system | Intestinal immune network for IgA production | 2 | 0.046128 |
| ko04115 | Cell growth and death | p53 signaling pathway | 2 | 0.053810 |
