# Supplementary material for: Chromosome-level genome assembly of grass carp (Ctenopharyngodon idella) provides insights into its genome evolution
Source: BMC Genomics. 2022 Apr 7;23:271. doi: 10.1186/s12864-022-08503-x (PMC8988418; doi:10.1186/s12864-022-08503-x)
Supplement: Supplementary file 14 — Additional file 14: Table S10. The top 20 statistically significant GO biological process terms of grass carp PSGs. [file 12864_2022_8503_MOESM14_ESM.docx]

| GO ID | Description | Count | *p* value |
| --- | --- | --- | --- |
| GO:0006955 | Immune response | 29 | 1.58e-16 |
| GO:0002376 | Immune system process | 32 | 3.91e-13 |
| GO:0016337 | Single organismal cell-cell adhesion | 13 | 2.24e-07 |
| GO:0098602 | Single organism cell adhesion | 13 | 2.71e-07 |
| GO:0002250 | Adaptive immune response | 8 | 1.55e-06 |
| GO:0098609 | Cell-cell adhesion | 14 | 2.55e-06 |
| GO:0006952 | Defense response | 14 | 4.63e-06 |
| GO:0007155 | Cell adhesion | 18 | 5.49e-06 |
| GO:0022610 | Biological adhesion | 18 | 6.02e-06 |
| GO:0002682 | Regulation of immune system process | 12 | 9.10e-06 |
| GO:0001775 | Cell activation | 11 | 1.01e-05 |
| GO:0046649 | Lymphocyte activation | 9 | 1.37e-05 |
| GO:0042110 | T cell activation | 8 | 1.38e-05 |
| GO:0070489 | T cell aggregation | 8 | 1.38e-05 |
| GO:0071593 | Lymphocyte aggregation | 8 | 1.45e-05 |
| GO:0070486 | Leukocyte aggregation | 8 | 1.59e-05 |
| GO:0007159 | Leukocyte cell-cell adhesion | 8 | 2.00e-05 |
| GO:0002684 | Positive regulation of immune system process | 9 | 2.26e-05 |
| GO:0022407 | Regulation of cell-cell adhesion | 7 | 2.41e-05 |
| GO:0034109 | Homotypic cell-cell adhesion | 8 | 2.60e-05 |
