# Supplementary material for: Chromosome-level genome assembly of grass carp (Ctenopharyngodon idella) provides insights into its genome evolution
Source: BMC Genomics. 2022 Apr 7;23:271. doi: 10.1186/s12864-022-08503-x (PMC8988418; doi:10.1186/s12864-022-08503-x)
Supplement: Supplementary file 15 — Additional file 15: Table S11. The top 20 statistically significant KEGG pathways of grass carp PSGs. [file 12864_2022_8503_MOESM15_ESM.docx]

| Pathway ID | KEGG class | Pathway | Count | *p* value |
| --- | --- | --- | --- | --- |
| ko04060 | Signaling molecules and interaction | Cytokine-cytokine receptor interaction | 24 | 1.78e-17 |
| ko04659 | Immune system | Th17 cell differentiation | 12 | 2.59e-08 |
| ko04658 | Immune system | Th1 and Th2 cell differentiation | 11 | 3.71e-08 |
| ko05340 | Immune diseases | Primary immunodeficiency | 7 | 7.51e-08 |
| ko04640 | Immune system | Hematopoietic cell lineage | 10 | 1.50e-07 |
| ko05162 | Infectious diseases | Measles | 11 | 2.22e-07 |
| ko04630 | Signal transduction | Jak-STAT signaling pathway | 11 | 3.05e-07 |
| ko05321 | Immune diseases | Inflammatiory bowel disease (IBD) | 8 | 1.43e-06 |
| ko04514 | Signaling molecules and interaction | Cell adhesion molecules (CAMs) | 13 | 1.97e-06 |
| ko05332 | Immune diseases | Graft-versus-host disease | 7 | 7.31e-06 |
| ko04940 | Endocrine and metabolic diseases | Type I diabetes mellitus | 7 | 2.55e-05 |
| ko05168 | Infectious diseases | Herpes simplex infection | 11 | 2.60e-05 |
| ko05330 | Immune diseases | Allograft rejection | 6 | 0.000102 |
| ko05416 | Cardiovascular diseases | Viral myocarditis | 7 | 0.000111 |
| ko05320 | Immune diseases | Autoimmune thyroid disease | 6 | 0.000142 |
| ko04672 | Immune system | Intestinal immune network for IgA production | 6 | 0.000184 |
| ko05166 | Infectious diseases | HTLV-I infection | 12 | 0.000195 |
| ko04650 | Immune system | Natural killer cell mediated cytotoxicity | 7 | 0.000259 |
| ko05142 | Infectious diseases | Chagas disease (American trypanosomiasis) | 7 | 0.000602 |
| ko05167 | Infectious diseases | Kaposi sarcoma-associated herpesvirus infection | 8 | 0.00228 |
