# Supplementary material for: Chromosome-level genome assembly of grass carp (Ctenopharyngodon idella) provides insights into its genome evolution
Source: BMC Genomics. 2022 Apr 7;23:271. doi: 10.1186/s12864-022-08503-x (PMC8988418; doi:10.1186/s12864-022-08503-x)
Supplement: Supplementary file 16 — Additional file 16: Table S12. Number of CNEs specific presence and deletion in one of the cyprinid genomes. [file 12864_2022_8503_MOESM16_ESM.docx]

| Genome | CNEs specific presence | CNEs specific deletion |
| --- | --- | --- |
| *D. translucida* | 3530 | 89346 |
| *C. idella* | 6710 | 1077 |
| *M. amblycephala* | 5714 | 650 |
| *O. macrolepis* | 4474 | 1026 |
| *C. carpio* | 4066 | 653 |
| *C. auratus* | 4080 | 883 |
| *S. rhinocerous* | 2029 | 142 |
| *S. anshuiensis* | 1852 | 165 |
| *S. grahami* | 1986 | 316 |
